# Exploring kinase family inhibitors and their moiety preferences using deep SHapley additive exPlanations

**DOI:** 10.1186/s12859-022-04760-5

**Published:** 2022-06-20

**Authors:** You-Wei Fan, Wan-Hsin Liu, Yun-Ti Chen, Yen-Chao Hsu, Nikhil Pathak, Yu-Wei Huang, Jinn-Moon Yang

**Affiliations:** 1 Institute of Molecular Medicine and Bioengineering, National Chiao Tung University, Hsinchu, 30050 Taiwan; 2 Institute of Bioinformatics and Systems Biology, National Chiao Tung University, Hsinchu, 30050 Taiwan; 3 Institute of Information Science, Academia Sinica, Taipei, 11564 Taiwan; 4 Bioinformatics Program, Taiwan International Graduate Program, Academia Sinica, Taipei, 11564 Taiwan; 5 Institute of Bioinformatics and Structural Biology, National Tsing Hua University, Hsinchu, 30044 Taiwan; 6 Institute of Biomedical Engineering, National Chiao Tung University, Hsinchu, 30050 Taiwan; 7 Department of Biological Science and Technology, National Chiao Tung University, Hsinchu, 30050 Taiwan

**Keywords:** Explainable deep neural networks, SHapley Additive exPlanations, Kinase family inhibitors, Moiety preferences, Common and specific moieties

## Abstract

**Background:**

While it has been known that human protein kinases mediate most signal transductions in cells and their dysfunction can result in inflammatory diseases and cancers, it remains a challenge to find effective kinase inhibitor as drugs for these diseases. One major challenge is the compensatory upregulation of related kinases following some critical kinase inhibition. To circumvent the compensatory effect, it is desirable to have inhibitors that inhibit all the kinases belonging to the same family, instead of targeting only a few kinases. However, finding inhibitors that target a whole kinase family is laborious and time consuming in wet lab.

**Results:**

In this paper, we present a computational approach taking advantage of interpretable deep learning models to address this challenge. Specifically, we firstly collected 9,037 inhibitor bioassay results (with 3991 active and 5046 inactive pairs) for eight kinase families (including EGFR, Jak, GSK, CLK, PIM, PKD, Akt and PKG) from the ChEMBL25 Database and the Metz Kinase Profiling Data. We generated 238 binary moiety features for each inhibitor, and used the features as input to train eight deep neural networks (DNN) models to predict whether an inhibitor is active for each kinase family. We then employed the SHapley Additive exPlanations (SHAP) to analyze the importance of each moiety feature in each classification model, identifying moieties that are in the common kinase hinge sites across the eight kinase families, as well as moieties that are specific to some kinase families. We finally validated these identified moieties using experimental crystal structures to reveal their functional importance in kinase inhibition.

**Conclusion:**

With the SHAP methodology, we identified two common moieties for eight kinase families, 9 EGFR-specific moieties, and 6 Akt-specific moieties, that bear functional importance in kinase inhibition. Our result suggests that SHAP has the potential to help finding effective pan-kinase family inhibitors.

## Background

Protein kinases play important regulatory roles in cellular signal transduction including apoptosis, cell cycle progression, cytoskeleton rearrangement, differentiation, development, immune response, nervous system function, and transcription [1, 2]. When kinase pathways are dysregulated, a variety of diseases occur, including diabetes and autoimmune diseases, inflammatory diseases, nervous disorders and cancer. Therefore, protein kinases have been one of the most important drug targets in recent years, accounting for a quarter of all current drug development working. Up to date, 61 small molecule protein kinase inhibitors have been approved by the US FDA [3]. Most of these inhibitors only target a few specific protein kinases. However, previous studies on cancer clinical treatment have pointed out that inhibiting only a single kinase can easily lead to compensatory upregulation of other cancer pathways, and in turn reduce the effectiveness of the cancer treatment [4, 5]. Besides, statistics from the protein–protein interactions networks have indicated that kinases that belong to the same family are highly co-regulated in related cancer pathway [6]. Hence, inhibition of a whole kinase family can significantly improve therapeutic efficacies. Yet, finding inhibitors that target a specific kinase through experimental profiling is time consuming and laborious in wet lab, and this is even more so if we are to find inhibitors that target a whole kinase family. An efficient drug screening strategy for identifying pan-kinase family inhibitors will be a great contribution to drug discovery and the treatment of cancers and inflammatory diseases.

We present in this paper a data-driven deep learning approach that uses explainable deep neural networks to address this issue. Specifically, we derive a new kinase-inhibitor bioactivity dataset and use deep neural networks (DNN) to predict whether an inhibitor is active for each of the eight kinase families under consideration. We note that the deep learning methodology has been employed in chemo-informatics and medicinal chemistry to predict the efficacies of new active small molecule inhibitors. Research has also been done using random forest and DNNs for inhibitor prediction for single kinase [7–10], and convolutional neural networks (CNNs) for protein–ligand binding affinity prediction [11]. For example, Rodríguez-Pérez and Bajorath [9] showed that a DNN with three layers performs slightly better than alternative tested machine learning methods including support vector machine and random forest, for ligand-based prediction of the activity of 19,030 ligand compounds against 103 kinases. But, to the best of our knowledge, using DNNs to predict inhibitors for whole kinase families has not been attempted before.

While deep learning can lead to accurate classifiers, deep learning models are often regarded as “black boxes” because of their high complexity, making interpretation of the model results difficult [12]. This limits the practical applicability of deep learning in drug discovery research. For example, Vamathevan et al. [13] discussed the issue that a typical issue with deep-trained neural networks is model interpretation and extraction of biological insights. To make the result interpretable, the use of explainable DNNs for the prediction of kinase inhibitors has been recently explored in [14, 15], both using the SHapley Additive ExPlanations (SHAP) method, a game theoretic approach that represents the state-of-the-art approach for explaining the output of machine learning models [16]. Our work extends these existing work in that we build explainable DNNs for whole kinase families. We show that, with SHAP, we can quantify the contribution of each moiety feature of the inhibitors for the classification tasks, and in turn identify moieties that are more often used in designing inhibitors of the same kinase family. These moieties are called *preference moieties* in this paper.

The major task of this paper is therefore the construction of an interpretable DNN classification model for kinase family inhibitors. This involves: (i) Building a novel kinase family inhibitor bioactivity dataset for the DNN model, (ii) Identifying 34 moieties and 204 Checkmol fingerprints as features for the inhibitors, (iii) Creating eight DNN models, one for each of the eight kinase families, and (iv) Inferring the preference moieties of inhibitors for each kinase family and the common moieties of inhibitors for all the eight kinase families using SHAP. We demonstrate that our approach can provide an efficient strategy for identifying and designing selective inhibitors targeting pan-kinase families.

## Results

### Kinase family inhibitors and model performance evaluation

We trained our DNN models to take the 238 moiety features of a compound as the input to predict whether the compound is active for one of the eight kinase families. We randomly divided the data of each kinase family into 80% training set and 20% validation set, and repeated the experiment for 100 times to get the average result. Table 1 shows the average accuracy of the DNN models. We can see that our DNN models obtain accuracy (ACC) higher than 0.90 for four out of the eight kinases families (i.e., EGFR, Jak, PIM, and Akt). The ACC is higher than 0.75 for all the eight families. Among them, the prediction for the EGFR family in the TK group seems the easiest, reaching ACC 0.93, area under the receiver operating characteristic curve (AUC) 0.96, and Matthews correlation coefficient (MCC) 0.85. We note that the accuracy of the DNN models is positively correlated with the total number of inhibitors per kinase families: those reaching ACC higher than 0.90 all have more than 1,000 total inhibitors (see Table 2). Therefore, it may be possible to further improve the accuracy of the DNN models if more data become available in the future.

**Table 1:** Performance of DNN models for predicting eight datasets of kinase family inhibitors

Group	Family	Test set			*r*^*b*^
		ACC (avg.)^*a*^	AUC (avg.)^*a*^	MCC (avg.)^*a*^	
TK	EGFR	0.93	0.96	0.85	0.72
	Jak	0.92	0.96	0.83	0.45
CMGC	GSK	0.79	0.77	0.45	0.62
	CLK	0.75	0.74	0.37	0.12
CAMK	PIM	0.91	0.95	0.82	0.75
	PKD	0.85	0.68	0.28	0.18
AGC	Akt	0.93	0.98	0.85	0.86
	PKG	0.88	0.82	0.47	0.52

^a^The average performance of predictions by 100 models on validation sets

^b^The Pearson’s correlation coefficient between the SHAP scores and the odds ratios of the top 15 moiety features for each kinase family (refer to Methods: Features of compound datasets)

**Table 2:** The summary of the dataset of eight selected kinase family inhibitors

Groups	Kinase families (*f*)	No. of kinases (*M*_*f*_)	No. of active inhibitors	No. of inactive inhibitors	Total inhibitors	Ratio of active/inactive
TK	EGFR	4	692	809	1501	0.86
	Jak	4	1580	887	2467	1.78
CMGC	GSK	2	246	658	904	0.37
	CLK	4	178	419	597	0.42
CAMK	PIM	3	776	641	1417	1.21
	PKD	3	67	462	529	0.15
AGC	Akt	3	401	774	1,175	0.52
	PKG	2	51	396	447	0.13

Underlined—balanced datasets

Table 1 also shows that we have at least one family reaching ACC higher than 0.90 for three out of the four groups: EGFR and Jak (TK), PIM (CAMK), and Akt (AGC). The prediction for the two families under the group CMGC seems more difficult, which might be related to the relatively fewer number of inhibitors for these two families. Among the two, GSK performs slighter better, with ACC 0.79, AUC 0.77, and MCC 0.45.

### Model interpretation and moiety features of kinase family inhibitors

**Fig. 1:**
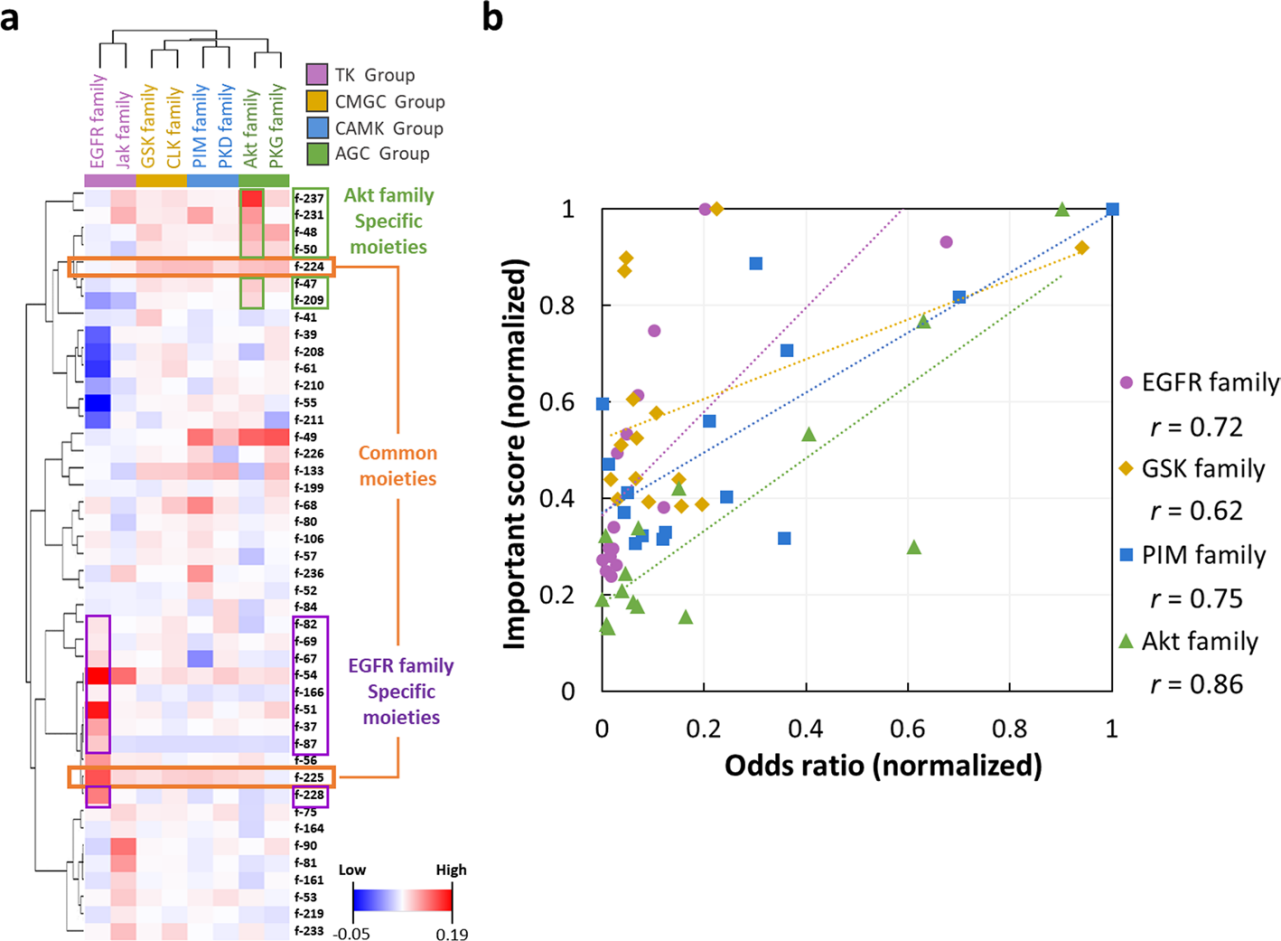
Clustering of features of eight kinase family inhibitors by Pearson’s $r$. **a** Hierarchical clustering using Pearson’s $r$ with the top 15 moiety features of each family, a combined total number of 44 moiety features. The color shed of the features indicate their SHAP importance score for each kinase family. Among the moiety features, we outline f-224 and f-225 in orange, for they have high SHAP scores for most of the eight families. We also refer to them as the *common* moieties. Moreover, the 9 family-*specific* moieties for EGFR are outlined in purple, and the 6 family-*specific* moieties for Akt are outlined in green. **b** The correlation coefficient between the features’ SHAP important scores and the odds ratios for four kinase families, one for each kinase group

We then used the SHAP methodology to assess the contribution and important score (see Eq. (5)) of each of the 238 moiety features for each DNN model. Based on the importance scores, we selected the top 15 moiety features with a z-score larger than 1.96, showing significant contribution for active inhibitor compound prediction, for each kinase family. We took the union of these selected moiety features across the eight families, leading to 44 moiety features in total, and performed Pearson’s hierarchical clustering of these features, to investigate the common and family-specific functional moieties. Result shown in Fig. 1a demonstrates that the kinase family inhibitors in the same kinase group have similar preference moieties and form clusters in the first level of the class hierarchy, which nicely aligns with our expectation. We can also see that the TK group seems to have quite different preference moieties than the other three groups.

To study whether the moieties selected by SHAP can indeed differentiate active and inactive inhibitors for different families, we calculated the Pearson's $r$ values between the SHAP important scores and the odds ratios of the eight kinase families. As shown in the rightmost column of Table 1, the correlation is higher than 0.40 for six out of the eight families, and we can find at least one family from each group that has correlation higher than 0.60 (namely EGFR, GSK, PIM and Akt; also see Fig. 1b). The highest correlation 0.86 is achieved by the Akt family. This result indicates that these top 15 moieties per kinase family can distinguish between active and inactive inhibitors.

In Fig. 1a, we also highlighted three groups of moieties. First, we consider f-224 and f-225, which have high SHAP values for most of the eight families, as the *common* moieties and highlight them in orange. Second, we regard nine moieties which have high SHAP values only for EGFR as *EGFR-specific* moieties and highlight them in purple. Similarly, we identify six *Akt-specific* moieties and highlight them in green. We only considered EGFR and Akt in the subsequence analysis of family-specific moieties, since the DNN models for these two families have the highest accuracy, and since the average importance scores (shed between blue and red) for these two families are relatively higher.

### Investigating the importance of the common & specific moieties in the crystal structures of protein–ligand complexes

We further validate the effectiveness of the SHAP analysis by checking whether moieties with high SHAP values do occur more frequently than moieties with low SHAP values in the crystal structures of protein–ligand complexes from two sources: the protein data bank (PDB), and our kinase family inhibitors set (i.e., what we compiled from ChEMBL25 and Metz Kinase Profiling Data; see Method). This can be done by calculating the percentage of times a moiety occurs in the ligand of a cocrystal structure of a kinase belonging to a certain kinase family. For each kinase family, we report the average percentage of occurrence of the following three groups of moieties: the *common* moieties, the *top-15* moieties per kinase family, and the *remainder* (i.e., the bottom-29 moieties). For EGFR and Akt, we can also report the average percentage of occurrence for the *family-specific* moieties.

**Fig. 2:**
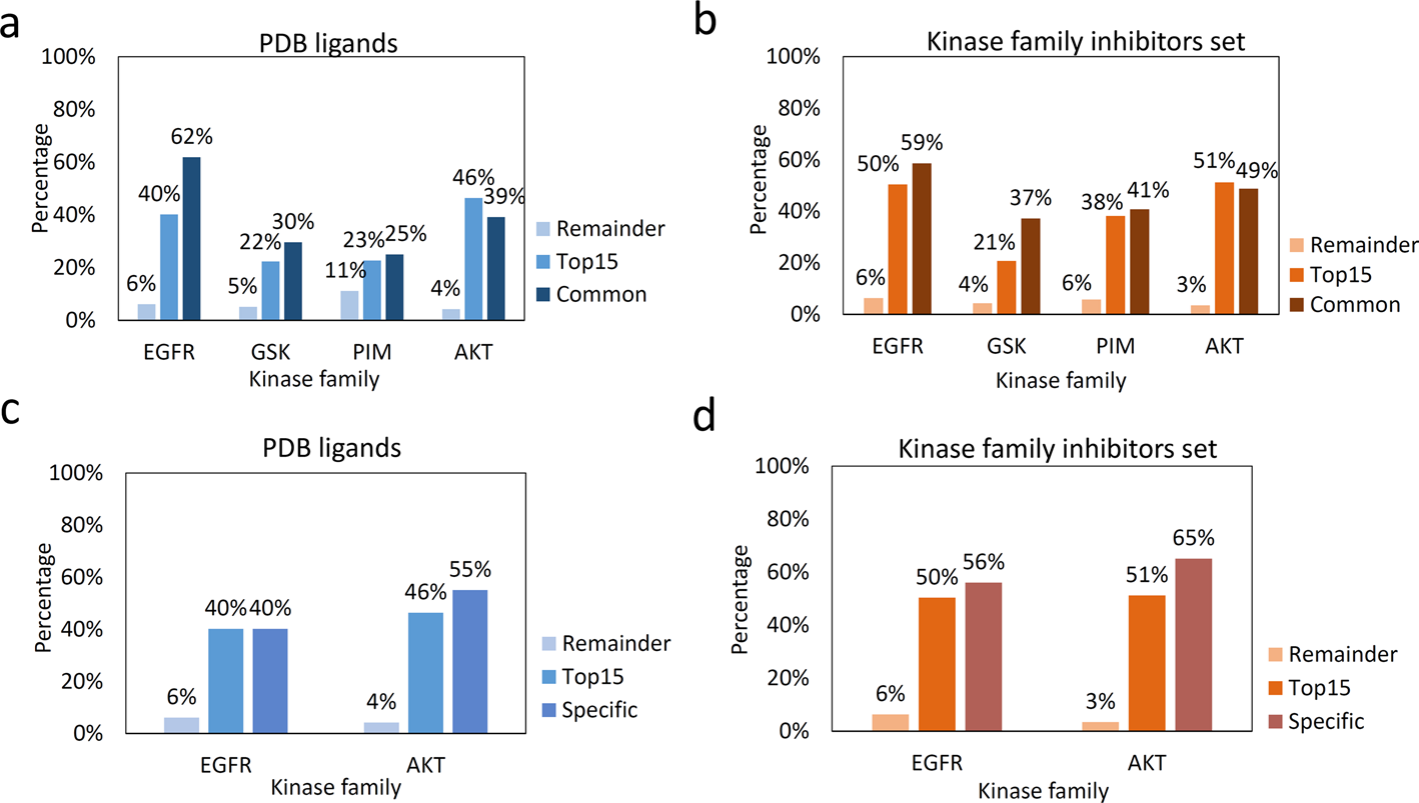
Average percentage of occurrence of different groups of moieties in the ligand of a cocrystal structure of a kinase belonging to a certain kinase family in the PDB and the kinase family inhibitors set. Subfigures **a** and **b** show the average percentage of occurrence of the two common moieties, the top-15 moieties per kinase family, and the bottom-29 moieties (notated as Remainder) for the 4 kinase families from different groups in PDB and Kinase family inhibitors sets, respectively. Subfigures **c** and **d** highlight the average percentage of occurrence of the family-specific moieties for the EGFR and Akt kinase families, in PDB and Kinase family inhibitors sets, respectively

Figure 2a, b show that in most cases, the average percentage of occurrence of the two common moieties are higher than the top 15 moieties and the *reminder* in all families’ datasets, except for the Akt family set where the common moieties is slightly lower than the top 15 moieties. For example, in the EGFR family, the average percentage of occurrence of the two common moieties is 62% in PDB ligands (Fig. 2a) and 59% in the kinase family inhibitors set (Fig. 2b), respectively.

Figure 2c, d report the average percentage of occurrence of the family-specific moieties for the inhibitors of the EGFR and Akt families. We see that the average percentage of occurrence of the family-specific moieties are also higher than the other top 15 moieties for these two kinase families in both PDB ligands and the kinase family inhibitors set. Comparing Fig. 2a and c, and comparing Fig. 2b and d, we see that, for the Akt family, the average percentage of occurrence of the common moieties is lower than the family-specific moieties (39% vs. 55% in Fig. 2a and c; and 49% vs. 65% in Fig. 2b and d).

**Fig. 3:**
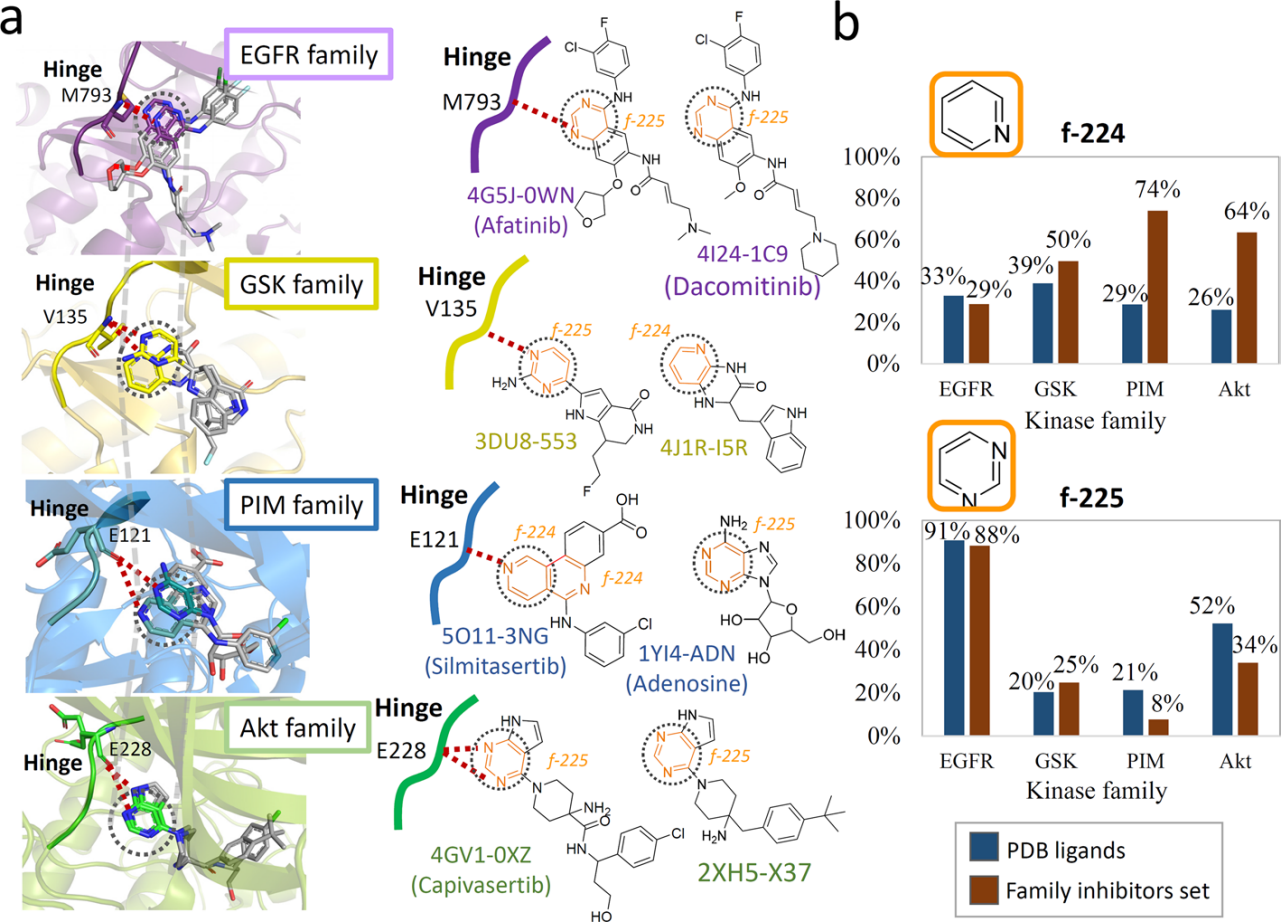
Common moieties in various kinase inhibitors from four different families. **a** *Common* moieties, f-224 (pyridine) and f-225 (pyrimidine), colored orange, interacting with the kinase hinge residues in the EGFR, GSK, PIM and Akt families. **b** Average frequencies of occurrence of *common* moiety in PDB ligands and kinase family inhibitors set

To further investigate the role the two *common* moieties in protein–ligand interactions, we respectively selected two protein-inhibitor cocrystal structures from each of the four kinase families to represent the possible interactions between ligand and protein of each kinase groups. By retrieving these cocrystal structures from the PDBsum database (http://www.ebi.ac.uk/pdbsum), we found most of these moieties are involved in Hydrogen bond Interactions or van der Waals with the hinge region residues of kinase as depicted. For example, the N-atom of f-225 moiety of Afatinib and Dacomitinib usually form an H-bond with the backbone amide group of M793 hinge residue of EGFR. The similar interactions can also be observed in other ligand-kinase families as shown in Fig. 3a. These results of forming H-bond with the hinge residue may echo the structure similarity between the f-224 (pyridine), f-225 (pyrimidine) and adenine of ATP, because the adenine of ATP always form hydrogen bonds with the hinge region of kinases. In addition, the high frequency of moieties f-224 and f-225 in all the four kinase families further suggests that these two moieties play an irreplaceable role in the kinase inhibition (Fig. 3b). Generally speaking, the frequency of moiety f-224 in the family inhibitor sets is > 50%, except for EGFR. As for f-225, the moiety frequency in the EGFR family inhibitors is as high as 90% (in both PDB ligands and inhibitor sets), indicating that almost all of the inhibitors contain this moiety.

**Fig. 4:**
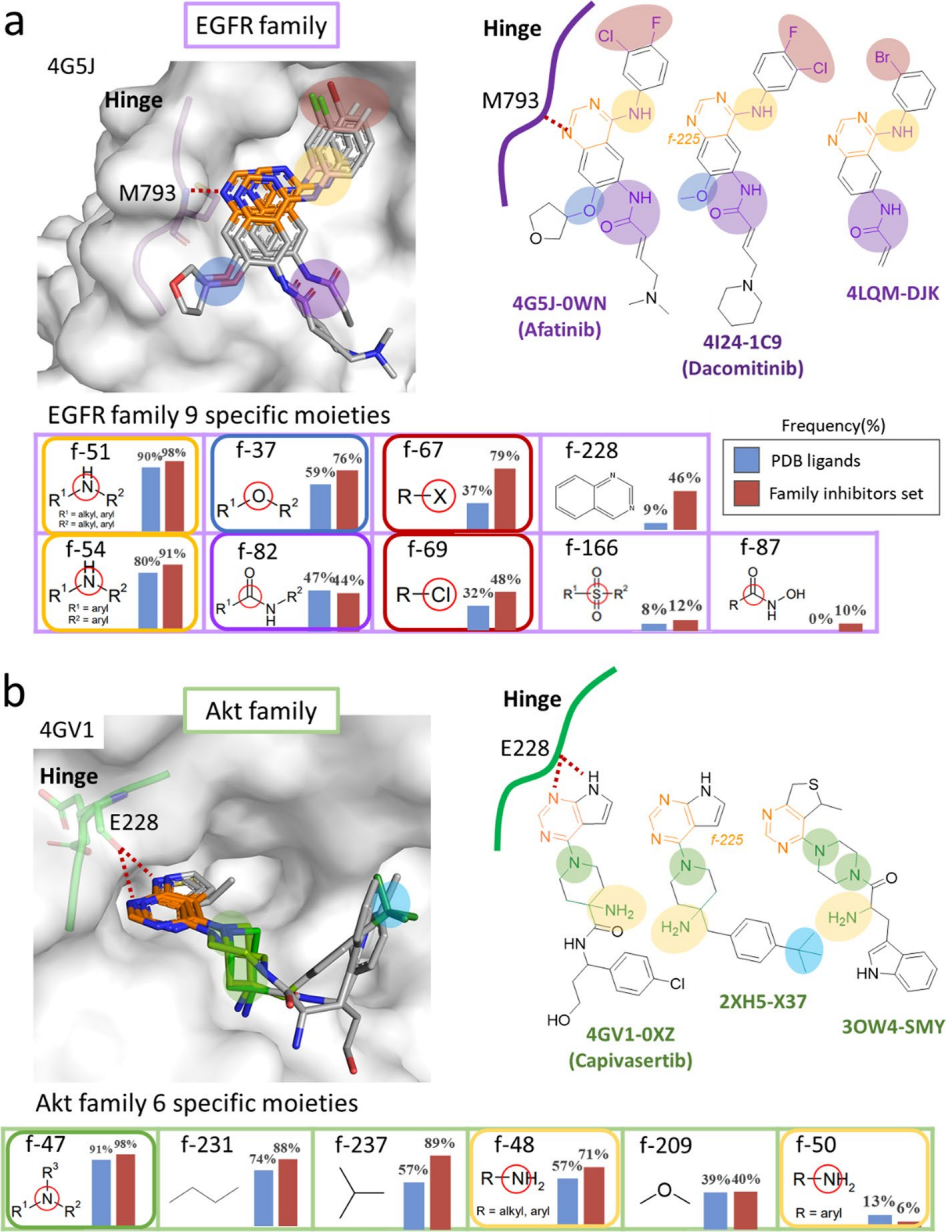
Family-specific moieties and moiety interacting preferences of the EGFR and Akt family inhibitors. **a** Nine EGFR-family specific moieties, the interaction residue M793 and three EGFR family inhibitors shown by bound structures (PDB codes: 4G5J, 4I24, and 4LQM as well as their bound ligands 0WN, 1C9, and DJK respectively). **b** Six Akt-family specific moieties and interacting residues like E228 with of three Akt family inhibitor bound structures (PDB codes: 4GV1, 2XH5, and 4LQM as well as their bound ligands 0XZ, X37, and SMY respectively)

We further proceed to investigate the role of *specific* moieties for each kinase family inhibitors as shown in Fig. 4. There are nine family-specific moieties observed in the inhibitors of the EGFR family (Fig. 4a), but only seven of them locate in the kinase subpockets. A secondary amine (f-51 and f-54) near the *common* moiety, an ether group (f-37) for hydrophobic interactions [17], two halogens on the aromatic ring (f-67 and f-69), and an amide group (f-82 and f-87) near the alkenyl group. After literature survey, we inferred that most of these moieties are designed to help anchor the *common* moieties at ATP-binding site, and do not directly participate in the substrate binding. For example, the amide group are introduced to form an enamide. By undergoing the Michael addition reaction, this enamide and Cys797 of EGFR can form a covalent adduct [18]. In addition, the halogen group usually interacts with the αC helix of the kinase [19] and the ether group is for interacting with Leu residue of the kinase. The two richest family-specific moieties, secondary amine, formed could be possibly due to the synthetic procedure starting with an adenine-like structure [20].

However, some of these family-specific moieties are necessary for substrate binding. Take the inhibitors of Akt family as an example, we see six family-specific moieties of Akt inhibitors are found in Fig. 2b. We noticed that the high frequency of amine moiety (f-47) or the amino group (f-48 and f-50) appearing in the specific moieties. After retrieving the detailed cocrystal structure data from the PDBsum database, we found the main reason for the necessity for the amino group might be due to the need of forming H-bonds with active site residues E234 (also known as E236), and E278 (also known as E279) [21]. These two residues and E228 are known for forming H-bonds with its substrate while phosphorylation.

## Discussion and conclusion

Our work represents a first attempt that explores the use of explainable deep learning models for finding the moiety preferences of kinase family inhibitors. In doing so, we compiled a new dataset of high-confidence kinase-inhibitor bioactivity data for eight kinase families. We constructed DNN models that can distinguish between active and inactive compounds for different kinase families with > 90% accuracy, and employed the SHAP methodology to access the importance of the moiety features of the compounds in making the model prediction. We showed that the SHAP scores enable us to identify common and family-specific moieties, by validating the importance of these moieties through their inhibitor complexed kinase PDB structures.

We note that it would have been better if our computational approach can be applied to a more comprehensive set of kinase families in our experiments, and preferably with more kinases per family. The experiments presented in this work concerns with only eight kinase families, and two of them only have two kinases per family, which is clearly small. This limitation comes from the fact that most kinases in either the ChEMBL25 Database or the Metz Kinase Profiling Data do not have complexed kinase PDB structures available for analysis, so we had to filter out these kinases. Future work is needed to address this issue and expand the analysis reported in this paper.

Moreover, in our experiments we mostly verify the importance of the identified moieties through discussions on their PDB structures. This can be extended with empirical evaluation of the effectiveness of the related compounds in kinase inhibition in web lab, for example by using compounds do not have those moieties. This can be an important future work.

Because there is a corresponding relationship between each kinase and inhibitor, it would also be interesting to include features of corresponding kinase in future work. For example, we may use the deep-learning based method described in [22] to extract kinase features. However, most of the selected compounds in our dataset do not have corresponding cocrystal structures with the targeted kinases. Due to this limit, we consider only features extracted from the compound in this work and leave the exploration of kinase features to future work.

We note that it has been found by different groups of researchers that multi-task DNN models tend to lead to higher classification accuracy than single-task DNN models [9, 23]. We consider only single-task DNN models in this paper for simplicity, building one binary classifier for each kinase family. We leave the exploration of more advanced network architectures (including multi-task DNN) as future work. Moreover, as our goal is to interpret what the DNN models learn, rather than pursuing high classification accuracy, we do not adopt other machine learning methods to build the classifiers. We refer readers to [7, 9, 24] for such performance comparisons.

In sum, in view of with the rapid development of deep learning based machine learning models, we believe the application of such models, especially those that are interpretable, to machine learning guided computational drug discovery a promising research direction. We hope this work can inspire more such endeavors, and more specifically contribute to improve virtual or high-throughput screening that in turn improves the development efficiency and hit rate of kinase-family inhibitors and similar drugs.

## Methods

### Overview

**Fig. 5:**
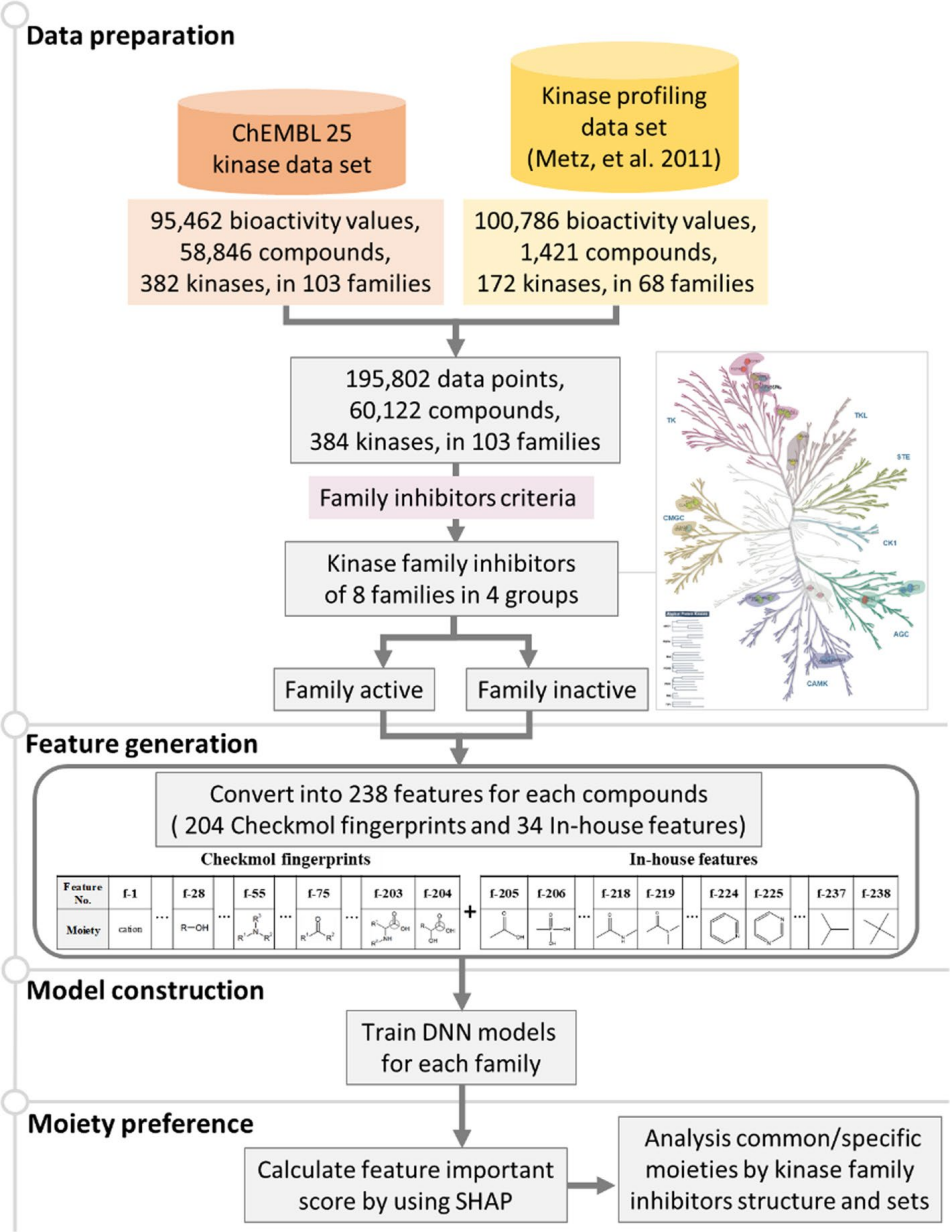
The framework of our research. The ChEMBL25 Dataset consists of 58,846 compounds, 382 kinases, and 95,462 kinase-compound bioactivity data points. The Metz Kinase profiling dataset contains 1421 compounds, 172 kinases, and 100,786 bioactivity data points [26]. Then, statistic criteria are used to select compounds that can be regarded as efficient inhibitors for kinase family. This selection process leads to inhibitors that cover eight kinase families. For each compound, we generated 238 moiety-based features for constructing DNN models for these kinase families. The SHAP approach is applied to uncover the preference moieties of each kinase family

Figure 5 shows a flowchart of how we constructed the DNN models to predict whether an inhibitor is active for a kinase family. First, we collected 196,248 kinase-compound pairing bioactivity data from two existing data sets, the ChEMBL25 Database [25] (with 95,462 bioactivity data points for 58,846 compounds and 384 kinases) and the Metz Kinase Profiling Data [26] (with 100,786 bioactivity data points for 1,421 compounds and 172 kinases). From these two data sets, we considered a compound as an efficient inhibitor for a whole kinase family based on several statistical criteria. We eventually selected inhibitors for eight kinase families, which are from 4 kinase groups. To analyze the moieties of these kinase family inhibitors, we generated 238 binary features for each compound, including 204 Checkmol fingerprints [27] and 34 manually-selected moieties. The vector of 238 features representing each compound would serve as the input to our DNN models. We built eight DNN binary classification models in total, one for each kinase family. ACC, AUC, and MCC were used to evaluate the performance of the DNN models on the validation sets. We utilized SHAP to interpret each DNN model and to infer the importance of each moiety of the kinase family inhibitors. Finally, we validated the derived moieties of kinase family inhibitors by kinase-inhibitor crystal structures.

### Preparation of datasets of inhibitors against a kinase family

We first collected kinase-compound bioactivity data sets from ChEMBL25 (https://www.ebi.ac.uk/chembl/) [25]. Specifically, we used the UniProt IDs [28] of 520 human kinases to retrieve kinase-compound bioactivity data points according to the following rules: (1) the bioactivity value is reported in “IC_50_”, i.e. the molar concentration of an agonist or antagonist which produces 50% of its maximum possible inhibition in a functional assay; (2) the standard activity relations are not unknown or not missing; (3) the type of assay relationship is “B”, i.e. binding assay; (4) the confidence score of assay, which is in [0, 9], reaches the highest value “9”, which means the compound has been assigned to a single protein with confidence. This process retrieves in total 95,462 kinase-compound pairing bioactivity data points, including 58,846 compounds for 382 kinases in 103 kinase families. For each bioactivity data point, we labeled it as *positive* when the IC_50_ is less than 500 nM; otherwise, we labeled it as *negative*.

The second data set we used is Metz Kinase Profiling Data [26]. After filtering the blank data points, Metz Kinase Profiling Data comprised 100,786 bioactivity values of 1,421 compounds against 172 kinases in 68 kinase families. In this data set, the bioactivity value is reported in *pKi*, i.e. the negative logarithm of the Ki value; the Ki value is the inhibition constant for a ligand, which denotes the affinity of the ligand for a receptor. For each bioactivity data point, we labeled it as *positive* when the *pKi* is greater than 6.3; otherwise, we labeled it as *negative*.

Combining ChEMBL25 and Metz Kinase Profiling Data, we may have multiple bioactivity data points for the same kinase-compound pair. We used the following voting mechanism to determine whether the compound is an *active* inhibitor for a kinase. If more than 80% of the recorded bioactivity values of this kinase-compound pair (from the union of the two datasets) are labeled as positive, we considered this compound as an *active* inhibitor for the kinase. Moreover, if less than 20% of the recorded bioactivity values are labeled as positive, we considered this compound as an *inactive* inhibitor for the kinase. This led to a final kinase-inhibitor set consisting of 195,802 unique bioactivity data points, which include 60,122 active inhibitors against 384 kinases in 103 families.

In the last step, we went one step forward and found inhibitors that are effective against a whole kinase family. Specifically, we considered a compound as an *active kinase family inhibitor* against a certain kinase family (labeled 1), if it can inhibit half of the members (M_*f*_) of that kinase family. Otherwise, we considered it as an *inactive kinase family inhibitor* (labeled 0). In addition, to make sure we have sufficient number of data per kinase family for training the DNN models, we selected kinase families according to the following criteria: (1) with 250 or more kinase family inhibitors, (2) with 2 or more kinases belonging to the family, (3) the kinases belonging to the family have crystal structures available from the PDB (http://www.rcsb.org/pdb/). This selection process yields inhibitors that against eight kinase families, which correspond to 4 kinase groups for further study. Each kinase family inhibitor set consists of at least 250 inhibitors and more than two kinases with crystal structures for validation. See Table 2 for a summary of the resulting dataset.

### Features for compound datasets

**Fig. 6:**
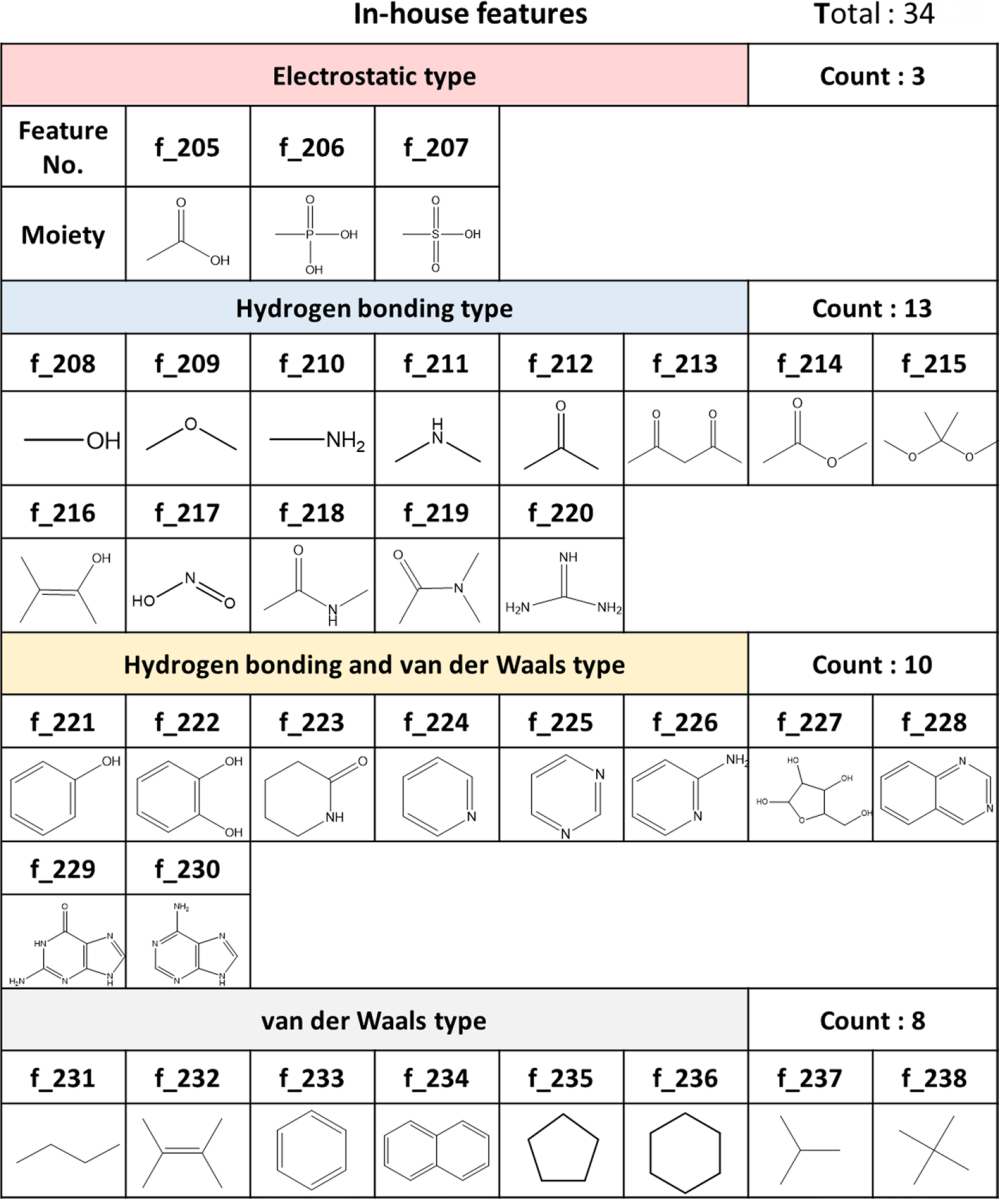
The 34 moieties we employ in addition to the Checkmol fingerprints for constructing the feature representations for the compounds. These include four types, namely electrostatic (3 moieties), hydrogen bonding (13 moieties), hydrogen bonding and van der Waals (10 moieties), and van der Waals type (8 moieties)

For each compound, 238 moiety-based molecular fingerprints, including 204 Checkmol fingerprints and 34 moieties, were used in the DNN models. Checkmol provides a comprehensive set of 204 binary statistical values derived from a given molecule, including the number of atoms, bonds, the number of C=O double bonds, and so on (https://homepage.univie.ac.at/norbert.haider/cheminf/cmmm.html) [27]. However, using Checkmol fingerprints is not enough, for Checkmol does not provide information regarding some pharmacophore ring structures in the compounds. To make up these deficiencies, we added 34 binary features describing the presence of specific ring-based substructures in the compound, as shown in Fig. 6. Among these moieties, 14 moieties were statistically inferred from 1382 FDA-approved drugs from DrugBank [29] and 6,163 biological metabolites from KEGG [30]. Also, 20 moieties are the common structures in 20 amino acids and kinase inhibitors. We can divide these features into four sub-groups based on the interaction types, including electrostatic, hydrogen-bonding, hydrogen-bonding & van der Waals, and van der Waals only types. Finally, these features were encoded as a 238-bit binary vector for the DNN models.

### Construction of deep neural networks

**Fig. 7:**
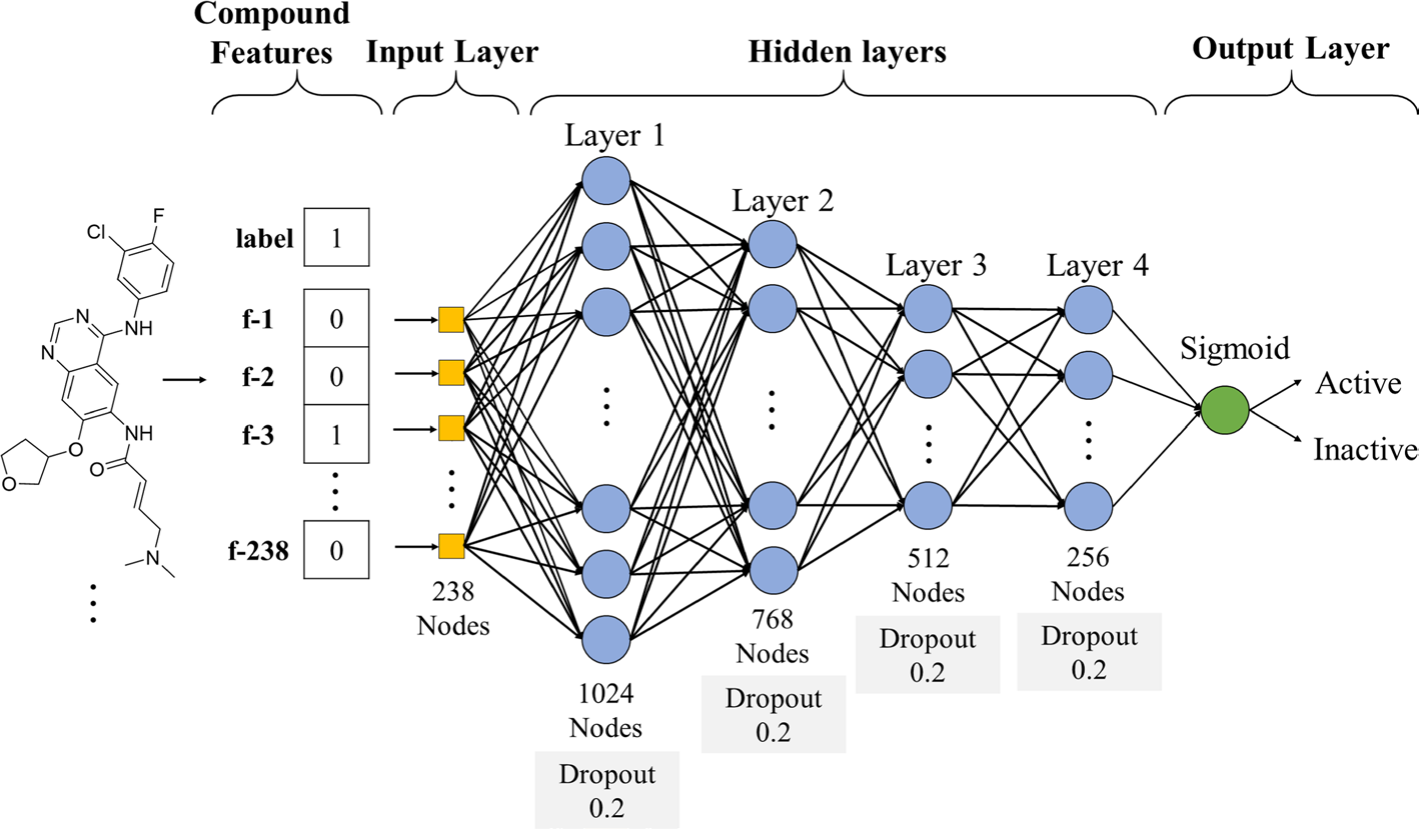
The DNN model framework for predicting kinase family inhibitors. The input layer includes 238 nodes which are the moiety features of an inhibitor. The four hidden layers comprise 1024, 768, 512, and 256 neurons (nodes), respectively. The output layer uses sigmoid to turn the model’s prediction into [0, 1], to make it a probabilistic estimate

Our DNN model [31] is composed of an input layer that takes the 238 moiety features, a number of hidden layers, and an output layer that makes binary classification, as illustrated in Fig. 7. The hidden layers learn on their own patterns from the input features that minimize a given loss function, with the following formula:

1 $${h}_{i,k}^{(l)}= \sigma \left({\sum }_{j=1}^{{M}^{(l-1)}}{h}_{i,j}^{\left(l-1\right)}{\omega }_{j,k}^{\left(l\right)}+{\omega }_{0,k}^{(l)}\right)$$

where ${h}_{i,j}^{(l-1)}$ denotes the output of the *j*-th neuron of the $\left(l-1\right)$-th hidden layer for the *i*-th training sample, ${M}^{\left(l-1\right)}$ denotes the number of neurons for the $\left(l-1\right)$-th hidden layer, $\left\{{\omega }_{0,k}^{(l)},{\omega }_{1,k}^{(l)},\dots ,{\omega }_{j,k}^{\left(l\right)}\dots ,{\omega }_{{M}^{(h-1)},k}^{(l)}\right\}$ denotes the (learnable) weights associated with the *k*-th neuron of the $l$-th hidden layer (where ${\omega }_{0,k}^{(l)}$ represents a bias term), ${h}_{i,k}^{(l)}$ denotes the output of this *k*-th neuron at $l$-th hidden layer for this training sample, and $\upsigma$ is an activation function that is implemented as a rectified linear unit (ReLU) [32]. We note that for $l$ = 1, namely the first hidden layer, we have ${h}_{i,j}^{(l-1)}={x}_{i,j}$, where ${x}_{i,j}$ denotes the *j*-th input moiety feature of the *i*-th training sample. We also note that the different hidden layers can have different numbers of neurons.

The parameters $\left\{{\omega }_{j,k}^{\left(l\right)}\right\}$ are learned by minimizing the following binary cross entropy [28] loss function using the training data:

2 $$-\frac{1}{N}\sum_{i=1}^{N}\left({y}_{i}log{\widehat{y}}_{i}+\left(1-{y}_{i}\right)log\left(1-{\widehat{y}}_{i}\right)\right)$$

where *N* denotes the number of training samples, ${y}_{i}\in \left\{0,1\right\}$ is the ground truth binary label for the *i*-th training sample, and ${\widehat{y}}_{i}\in \left\{0,1\right\}$ is the model’s predicted probability value. We note that the value of ${\widehat{y}}_{i}$ lies between 0 and 1 due to the final sigmoid layer at the output.

The aforementioned DNN architecture has many hyperparameters to be tuned, such as the number of hidden layers. In our work, we tuned the hyperparameters according to the classification accuracy on the validation sets for the EGFR kinase family. We chose EGFR here for its numbers of active and inactive inhibitors are large and relatively balanced. The search range for the hyperparameters is: the number of hidden layers: from 3 to 6, the number of neurons per layer: from 256 to 1,024, dropout rate (which helps avoid overfitting) [33]: from 20 to 50%, learning rate: from 0.001 to 0.00001, and batch size: 16, 32, 64. We employed Adam as the optimizer, and adopted batch normalization and dropout. After hyperparameter tuning, we finally decided to set the number of hidden layers to 4 (with number of neurons shown in Fig. 7), dropout rate to 20%, learning rate to 0.001 and batch size to 32.

### SHapley Additive exPlanations

SHapley Additive explanation (SHAP) is an extended application based on the Sharpe force value in game theory [34] and it represents the state-of-the-art approach for feature importance analysis in machine learning models [16]. It was originally developed to evaluate the importance of a single participant in a collaborative team, considering the average of all contributions made by participants. In the context of machine learning, SHAP considers each feature as a “participant” and treats the contribution of a feature toward the final decision of the machine learning model as the importance of that feature. If the SHAP value of a feature is positive, we know that feature is helpful for model prediction.

Assuming that the input of a machine learning model *f*, a DNN in our case, is a vector of *M* features, ${{\varvec{x}}}_{{\varvec{i}}}=\left({x}_{i,1},{x}_{i,2},\dots ,{x}_{i,M}\right)$, and denoting the model prediction as $f({{\varvec{x}}}_{{\varvec{i}}})$, SHAP entails establishing the following explanation model *g*:

3 $$f\left({{\varvec{x}}}_{{\varvec{i}}}\right)=g\left({{\varvec{x}}}_{i}^{^{\prime}}\right)={\phi }_{0}+\sum_{j=1}^{M}{\phi }_{j}{x}_{i,j}^{^{\prime}}$$

which is a simple linear function of ${x}_{i,j}^{^{\prime}}\in \left\{\mathrm{0,1}\right\}$, the binary version of ${x}_{i,j}$. Here, ${\phi }_{0}$ denotes the average output of the model *f* and therefore serves as a constant, and ${\phi }_{j}$ represents the desired contribution value of the *j*-th feature (a moiety feature in our case) for this particular input.

The contribution value ${\phi }_{j}$ of each feature is calculated by considering all possible combinations of features, with or without that *j*-th feature. This can be mathematically described as:

4 $${\phi }_{j}=\sum_{S\subseteq {x}^{^{\prime}}\backslash \{j\}}\frac{\left|S\right|!\left(M-\left|S\right|-1\right)!}{M!}[{f}_{x}\left(S\cup \left\{j\right\}\right)-{f}_{x}(S)]$$

where $S$ represents all possible subsets of the *M* features excluding the *j*-th, ${f}_{x}(S)$ is the model prediction when using *S* as the input feature (i.e., masking all the others not in *S*), and ${\mathrm{f}}_{\mathrm{x}}(\mathrm{S}\cup \{\mathrm{j}\})$ is the model prediction when the *j*-th feature is included.

We note that ${\phi }_{j}$ only represents the importance of the *j*-th feature for a particular input. To obtain the average contribution of the *j*-th feature across the training examples, we take the labels into account and use the following formula to get an aggregated importance estimate of the *j*-th feature:

5 $$Important \, scor{e}_{j}=avg.\left(\sum_{\left(act\right)=1}^{O}{\phi }_{j(act)}\right)-avg.\left(\sum_{\left(inact\right)=1}^{P}{\phi }_{j(inact)}\right)$$

where *O* and *P* denotes the number of active and inactive inhibitors for the target kinase family. Intuitively, the larger the importance score, the better the ability of this feature is in distinguishing between active and inactive compounds.

### Performance evaluation metrics

Before we use a DNN model for importance analysis, we need to quantify how accurate the model is in distinguishing between active and inactive kinase family inhibitor for a given kinase family. In doing so, we employed the following three performance metrics: ACC, AUC, and MCC defined as follows:

6 $$ACC=\frac{TP+TN}{TP+FN+FP+TN}$$

7 $$AUC=\frac{1}{2}(TPR+FPR)$$

8 $$MCC=\frac{TP\times TN-FP\times FN}{\sqrt{(TP+FP)\times (TP+FN)\times (TN+FN)\times (TN+FP)}}$$

where TP denotes true positive, TN true negative, FP false positive, and FN false negative. ACC is the ratio of correctly classified inhibitors in the data set. AUC is used to assess the capacity of the models to separate actives from inactive inhibitors. MCC is the correlation coefficient between the actual label and the predicted binary classification label.

## Data Availability

The original data used during the current study are available from the following two sources, ChEMBL25 (https://www.ebi.ac.uk/chembl/) and Metz Kinase Profiling Data (https://www.nature.com/articles/nchembio.530; see the “Supplementary information” Section). The processed data are available from the corresponding author on reasonable request.

## Abbreviations

DNN: Deep neural networks
SHAP: SHapley Additive exPlanations
CNN: Convolutional neural network
ACC: Accuracy
AUC: Area under the receiver operating characteristic curve
MCC: Matthews correlation coefficient
PDB: Protein data bank
ReLU: Rectified linear unit
